# A Brief Comment on Vasa Vasorum of Human Saphenous Vein: relevance for Coronary Artery Bypass Surgery

**DOI:** 10.21470/1678-9741-2020-0066

**Published:** 2021

**Authors:** Andrzej Loesch, Michael Richard Dashwood

**Affiliations:** 1 Centre for Rheumatology and Connective Tissue Diseases, University College London Medical School, Royal Free Campus, London, United Kingdom.; 2 Division of Surgery and Interventional Science, University College London Medical School, Royal Free Campus, London, United Kingdom.

**Keywords:** Saphenous Vein, Vasa Vasorum, Coronary Artery Bypass, Microvessels, Cardiovascular System, Femoral Vein

## Abstract

The importance of the vasa vasorum and blood supply to the wall of human saphenous vein (hSV) used for coronary artery bypass grafting (CABG) is briefly discussed. This is in the context of the possible physical link of the vasa vasorum connecting with the lumen of hSV and the anti-ischaemic impact of this microvessel network in the hSV used for CABG.

**Table:** 

Abbreviations, acronyms & symbols	
ar	= Arterioles
CABG	= Coronary artery bypass grafting
hSV	= Human saphenous vein
NA	= Noradrenaline
NT	= No-touch
PVAT	= Perivascular adipose tissue
SV	= Saphenous vein
ve	= Venule

## INTRODUCTION

The importance of the vasa vasorum in the performance (patency) of human saphenous vein (hSV) used for coronary artery bypass grafting (CABG) has been highlighted in various publications over the last 15 years^[1-9]^. It has been pointed out that the vasa system not only plays a role in delivering oxygen and nutrients to the vein wall - as it is generally accepted - but it also may act as a novel 'transport system'^[10]^. Principally the vasa vessels, including those located in the adventitia and those associated with the vein's surrounding pedicle of fat^[11]^, protect the hSV once implanted as grafts during CABG. A prime focus of this mini-review is on the luminal aspect of hSV and a possible link, either via tributaries or by direct contact, with the vein's rich vasa vasorum (venarum) system as elegantly presented previously^[3,4,12,13]^.

### Are there Vasa Openings into the Saphenous Vein Lumen?

One of the intriguing questions regarding blood supply through the wall of the hSV is whether there are direct openings (terminations) of the vasa vasorum into the vein lumen? This phenomenon was originally observed in the human femoral vein, examined with the use of a scanning electron microscope, by Brook, over 50 years ago^[14]^. Such observations indicated the possibility of physical communication between the vasa vasorum and the lumen of the host vein. However, according to Brook^[14]^, no such communication between the vasa microcirculation and the lumen was observed in the case of hSV. Similar observations and doubt in the existence of such direct vasa openings to hSV lumen have also been expressed and broadly discussed by Kachlik et al.^[3]^. Here, we revisit some of the previous observations and discussions in an attempt to clarify this topic.

Studies of hSV with India ink showed that the nearest/closest vasa vessels to the vein lumen are at a distance of ~70-100 µm, suggesting that there are no direct vasa contacts and openings in the hSV lumen^[3]^. Also, no evidence of vasa openings at the hSV lumen was observed in those vein segments affected by hyperplasia. In such cases, however, the India ink labelled some of the vasa vessels adjacent to subintimal regions associated with hyperplastic changes^[3]^. According to Fernández-Alfonso et al.^[9]^, when India ink is injected through the lumen of hSV, *in vitro*, it can subsequently be detected within the vein wall - in the medial and adventitial vasa vasorum - as well as within the vein-associated pedicle of perivascular adipose tissue (PVAT). Hence, these results clearly imply continuity between the vein lumen and these vascular structures labelled by the India ink. However, it is difficult, or perhaps even impossible, to identify any structural continuity between these India ink-labelled vasa structures, as observed in a given plane of a standard histological circumferential or longitudinal section of the vein. Therefore, regardless of whether the India ink is injected via the adventitial or luminal aspect of hSV (including the no-touch [NT] preparations of hSV for CABG, where adventitia and PVAT are left intact and the vein is not distended^[15]^, the course of the India ink travel from the lumen and dense vasa vasorum system might still be unclear. For more details on India ink-stained hSV vasa vasorum, see Kachlik et al.^[3,4]^ and Fernández-Alfonso et al.^[9]^.

### Corrosion Casts of the hSV Vasa Vasorum

Vascular corrosion cast studies of hSV have revealed the structural complexity of the vein vasa vasorum system^[3,4,12,13]^. In these studies, which included hSV segments harvested for CABG as well as hSV specimens obtained after postmortem delay, no direct openings of vasa vessels to hSV lumen were reported. Kachlik et al.^[3]^ have argued that if such openings were indeed present, then signs of these structures (openings) would be visible in the vascular corrosion casts of luminal aspects of hSV; these would appear at least as "blind ending vessels with rounded tips" if the luminal openings of vasa were not completely filled by the cast resin. This is an important observation as the viscosity of cast resin is usually much higher than the viscosity of blood, so the vascular infiltration by cast resin could be minimal or none in some circumstances (*e.g.*, during vasoconstriction or at other obstructions). One would not expect to get "normal" flow of 'viscous' resin through vasa vasorum/microvessels in postmortem samples as this tissue is, by definition, dead.

### Existence *vs.* Non-existence of Vasa Direct Openings

**Fig. 1:**
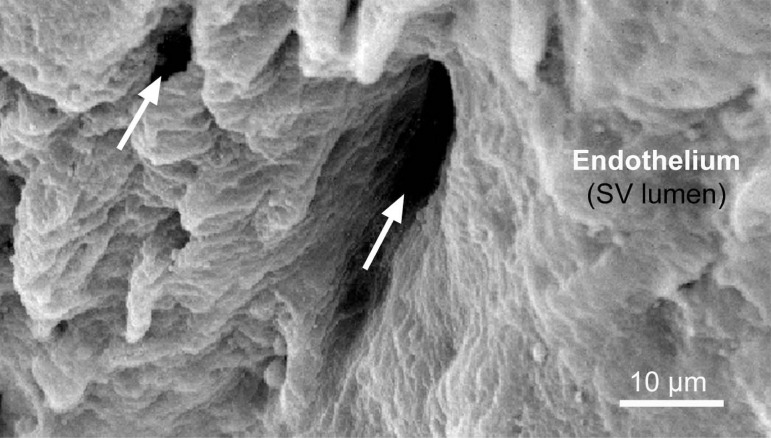
Scanning electron microscope (standard procedure) example of possible vasa vasorum/venarum direct openings into the lumen of the host vein - in this case of no-touch human saphenous vein (hSV) for coronary artery bypass grafting. Arrows point to the vasa openings of which one is < 10 µm of diameter, while the other is > 10 µm. Also note that the arrows indicate a possible direction of blood flow - from the vein lumen into the openings of vasa to be then delivered to the vein wall following vein implantation as coronary graft. For more details on the aspects of blood flow in hSV as coronary graft, see Loesch and Dashwood^[10]^, 2018. SV=saphenous vein

**Fig. 2:**
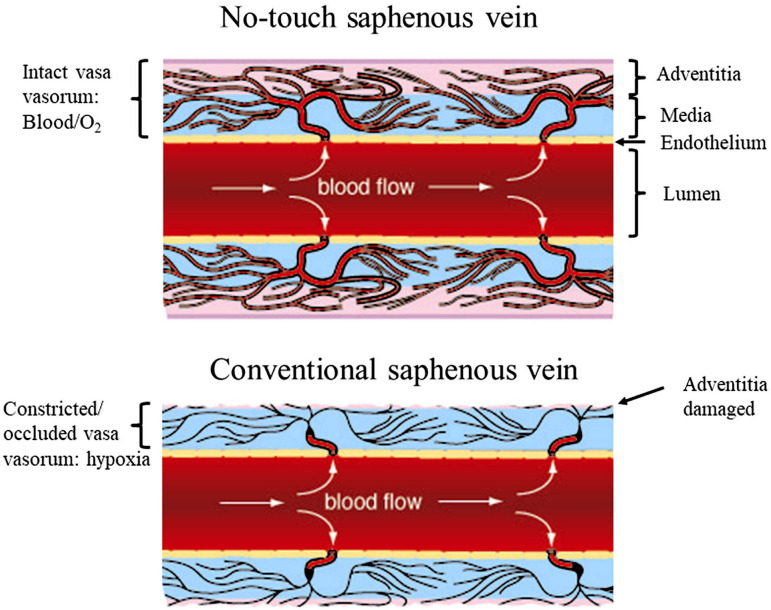
Diagrammatically proposed reduction in blood flow in damaged vasa vasorum (medial hypoxia) of human saphenous vein (hSV) harvested for coronary artery bypass grafting. Top panel: no-touch hSV where the adventitia and vasa vasorum are intact; blood flow to the vein wall is restored after graft implantation, oxygen and nutrient supply is maintained. Lower panel: Conventionally harvested hSV where the adventitia has been stripped off or damaged; the vasa vasorum is constricted or occluded, blood flow to the vein wall is reduced releasing hypoxic factors, which are associated with many aspects of graft failure. For more details, see Samano et al.^[22]^, 2015, and Loesch and Dashwood^[10]^, 2018. From an Open Access article: Loesch and Dashwood^[10]^, 2018, which is acknowledged.

Discussions regarding the existence *vs.* non-existence (or absence?) of direct openings of vasa at the hSV lumen are enriched by the results of elegant studies by Dr. Thomas P Crotty, who examined vasoregulatory function of the venous microcirculation using the canine lateral saphenous vein (SV), as well as skin flaps, as experimental models^[8,16-20]^. According to Crotty^[8]^, the direct vasa openings into host vein lumen can exist but are difficult to observe as they may be closed during smooth, non-turbulent, blood flow. In contrast, under turbulent flow or following stimulation with noradrenaline (NA), direct vasa openings can be seen at the intima of the host vein lumen. Such phenomena might explain, at least in part, the lack of findings of vasa openings (or "blind ending vessels") in corrosion casts of hSV^[3]^, where the vasa was subjected to non-turbulent perfusion with rather high (higher than that of blood) viscosity resin. Nevertheless, and in agreement with Crotty^[21]^, one has to recognise the significant structural and pharmacological differences between canine lateral SV and hSV. Again, and regardless of these differences, observations of the so-called NT hSV harvested for CABG (where there is no vein stripping and distension applied) reveal retrograde blood flow from the graft lumen to the adventitial vasa circulation, and where small calibre vessel-like openings of ~4 to 6 µm in diameter have been found in the graft luminal aspect^[6]^. The size of these vasa openings observed by Dreifaldt et al.^[6]^ therefore agrees with the report of vasa capillaries in hSV wall as small as 4.7 µm to 11.6 µm^[3]^. The observations by Dreifaldt et al.^[6]^ and Crotty^[8]^ clearly imply a functional role of such openings of vasa in the retrograde blood flow to the vein wall; importantly, this phenomenon also applies to the NT hSV when used as coronary grafts^[6]^. Here, Figure 1 demonstrates possible vasa vasorum direct openings to the lumen of hSV preparation for CABG as observed with the scanning electron microscope. And Figure 2 diagrammatically shows the state and consequences of damaged vasa vasorum in hSV harvested conventionally (where stripping and distention are applied) and conversely, the preserved, undamaged, vasa vasorum in NT hSV preparations for CABG.

It is therefore highly likely that the bulk of blood flowing to the vasa system in NT hSV grafts for CABG is via the vein tributaries, which remain present but are ligated some distance from the vein stem. This implies that the vasa vasorum of intact hSV in the lower limb may or may not contribute to blood flow to the vein lumen (via direct openings), while in NT hSV grafts for CABG, the blood from the lumen of the vein flows both via tributaries and via direct vasa openings to further supply the vasa microcirculation and the wall of venous graft^[6]^. In this scenario, tributaries and possible direct vasa terminations in NT hSV grafts for CABG now play a role of feeding vessels to local vasa vasorum^[10]^.

### How is this Happening: Vasa Openings and Turbulence?

The existence of small-sized direct openings, possibly linking vasa with the host vein lumen, does not necessarily mean that blood flow may occur in either direction at any time, namely from the vasa network into the vein lumen or vice versa. According to Crotty^[8]^, one of the factors that may influence the direction of flow is that of turbulence. This is the case because turbulent blood flow (or blood turbulence) introduces radial components/forces (*e.g.*, vortices), without which blood flow from the vein to its vasa microcirculation may not be possible. In theory, these radial components therefore act as "biological mini pumps" that distribute blood from the vein to its microcirculation/vasa^[8]^. Nevertheless, the previously mentioned retrograde flow and the direct connections between, *e.g.*, hSV lumen and the vein vasa vessels are in question. One argument presented by Kachlik et al.^[3]^ against the existence of such connections is an observation of red blood cells within the lumen of small calibre vasa vessels in hSV harvested for CABG^[6,23]^; according to Kachlik et al.^[3]^, such blood cells should not be present there after vein distention with saline (usually at pressure 300 mm Hg or more). However, the luminal pressure applied during saline distension (*n.b.*, not a saline perfusion) is distributed radially and possibly equally along the axis of hSV harvested for CABG. Therefore, there is no particular "dominating" saline flow vector, but rather a constant pressure is inflicted on the intima. Consequently, there is no movement and/or removal (wash out) of red cells from small calibre vasa vessels. In effect, red cells may remain stationary within vasa of non-leaking hSV preparations for CABG. It is important to stress that the stripping, and in particular high pressure distension, that is routinely applied to overcome spasm of conventionally harvested hSV for CABG causes distortion to the vein's original architecture, including the structure of the vein intima^[15,23]^. This may also distort and/or obscure direct small openings of vasa into the hSV lumen.

### Valve, Agger, Turbulence, and Vasa Vasorum

**Fig. 3:**
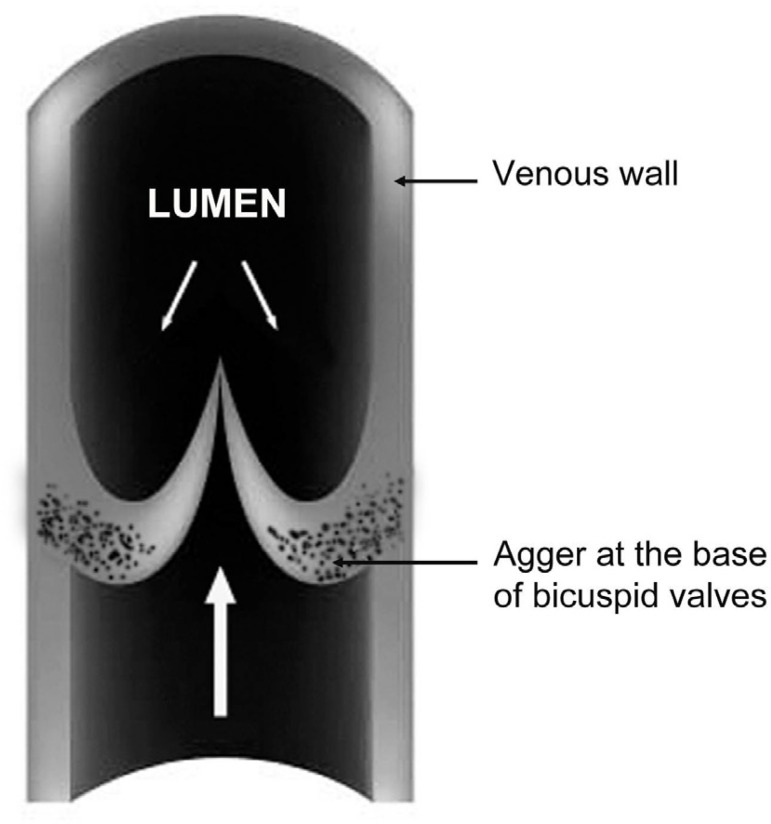
Diagrammatic representation of a longitudinal section through the vein (may be relevant to human saphenous vein) showing the venal bicuspid valves with their cusps/leaflets closed. Note that the agger is located at the base of valves. Large arrow indicates the main direction (against gravity) of blood movement, while the two small arrows, above valves, indicate a backflow of blood, in particular if valves are damaged in pathological conditions, and where the thickening of agger's structure (usually comprising smooth muscle and elastin) also occurs. For more details about venal valve and agger, see, e.g,. Corcos et al.^[31]^, 2000, and Crotty^[8]^, 2011.

Concerning the effects of turbulent flow, there is evidence of an impact of intact valves on the flow in reversed hSV used as infrainguinal vein grafts^[24]^. This phenomenon also concerns hSV preparations for CABG, where valves (reversed) cause luminal narrowing, thus creating disturbances in blood flow^[25-27]^. It should be pointed out that hSV has numerous valves irregularly distributed throughout the vein length^[28]^. According to Portugal et al.^[29]^, the average number of valves in hSV (taken from the medial epicondyle of the femur to the saphenous hiatus) is around 4.77-4.87; with a range of two to nine valves being present, all of which were bicuspid. Within hSV valves, at the base of the valve sinus, is located a "bizarre" structure, the agger, comprised mainly of smooth muscle and elastin^[20,30,31]^. Here, Figure 3 schematically shows the agger's location in the venal wall. In pathological conditions, such as varicose hSV with prolapsed valves, the agger's structure is enlarged due to thickened bundles of smooth muscle and the fragmentation and dissociation of the elastin membrane^[31]^. Apart from contributing to the control of blood flow in the vein lumen, healthy valves seem to also contribute to the vasa vasorum microcirculation system. This claim is supported by the observation of vasa vasorum openings at the base of the valve sinus located at tributary junctions, at least in the canine lateral SV^[8,18]^. According to Crotty^[19]^, the agger's structure is made up of bundles of smooth muscle cells and fibroelastin, with this structure playing a dual role: (i) in the movement of the valve; and (ii) in regulating blood flow between the vasa vasorum system and the lumen of the parental vein. The importance of blood flow between the vasa vasorum system and the lumen of the parental vein (please see sections above) is rarely discussed, in particular in the context of valve and agger's function. An exception seems to be the meticulous findings described by Crotty^[20,8]^. Accordingly, the agger is a crescentic, fibroelastic structure internal to the layer of circular smooth muscles of the vein wall; both the base of the agger and its apex (apex is located at the base of the valve sinus) rest on bundles of longitudinal smooth muscle cells. Consequently, when the venous tone rises, it constricts smooth muscles bundles of valve's agger. In effect, the agger's smooth muscles are pulling in opposite directions and the elastic fibres become stretched, which opens the lumen of local vasa vasorum venules that drain into the vein lumen. As an example, this has been observed in elegant studies of NA-elevated tone of canine lateral SV with injected India-ink^[17,18,20]^. Due to the structural features, including thickness, the role of valvular agger in human veins of lower limbs may also be to prevent local dilation of the vein ^[32]^. Interestingly, there appears to be a lack of attention regarding the role of the agger, including in the hSV, since there is no mention of this structure in medical textbooks, such as early editions of The Pathology and Surgery of the Veins of the Lower Limb^[33]^ or recent editions of the Gray's Anatomy.

**Fig. 4:**
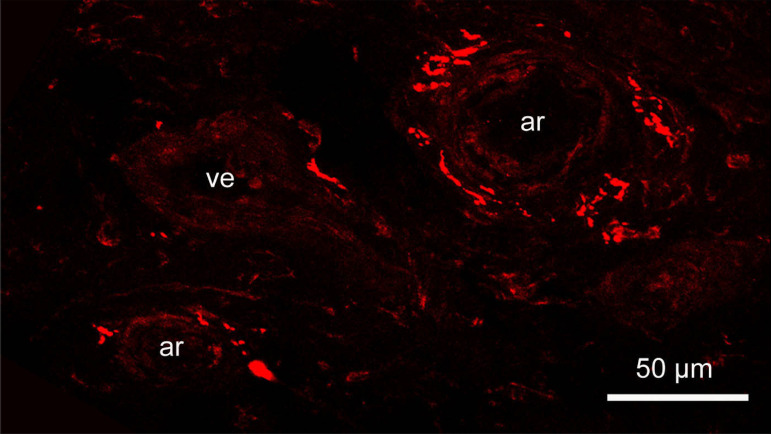
Confocal microscope image of no-touch human saphenous vein (hSV) (30 µm frozen section) immunolabelled (originally red) for tyrosine hydroxylase (TH; a marker for sympathetic nerves). A fragment of adventitia periphery showing TH-positive perivascular sympathetic nerves at small vasa vasorum blood vessels: two arterioles (ar) and one venule (ve). Bar: 50 µm. Note that a rabbit TH polyclonal antibody (TZ 1010, Affinity, Exeter, United Kingdom) was used at 1:300; goat antirabbit Alexa 568 (Molecular Probes, Oregon, United States of America) was used at 1:600 as a second layer; confocal laser microscope: BioRadiance 2000. For more details on the sympathetic innervation of hSV, see Loesch and Dashwood^[5]^, 2009.

In this mini-review, we have not discussed additional phenomena influencing physiology of the vasa vasorum such as the impact of the rich supply of nerves, in particular the sympathetic innervation of the hSV^[5]^ (Figure 4). However, it is known from Crotty's elegant studies that sympathetic nerves have a regulatory function in relation to (i) venous vasa vasorum microcirculation; (ii) valve and agger's function; (iii) mediating and/or competing with plasma NA; and finally (iv) the origin of varicose veins^[8]^. The extent to which Dr. Crotty appreciated the influence of sympathetic nerves and/or NA become clear on reading the final sentence of the Abstract in his last paper^[34]^: "Death is believed to occur when plasma noradrenaline has damaged the structure of the sympathetic system so much that it can no longer create the minimum quantity of neurotransmitter needed to maintain the level of noradrenergic balance and homeostasis necessary for life."

## CONCLUSION

Histological studies (*e.g.*, using India ink, the pharmacology of NA, and those with scanning electron microscope) support the hypothesis that there are luminal openings/terminations of vasa in the lumen of hSV (NT hSV) used for CABG. Notably, the occurrence of blood flow through the vasa of NT hSV used as grafts during CABG is the strongest indication, yet that there is a direct link (physical communication) between the hSV lumen and the vein vasa vasorum microcirculation. This phenomenon may have a physiologically protective anti-ischemic effect on the graft, particularly in its early stages after implantation into the coronary circulation.

**Table:** 

Authors' roles & responsibilities	
AL	Substantial contributions to the conception or design of the work; drafting the work or revising it critically for important intellectual content; agreement to be accountable for all aspects of the work in ensuring that questions related to the accuracy or integrity of any part of the work are appropriately investigated and resolved; ﬁnal approval of the version to be published
MRD	Substantial contributions to the conception or design of the work; drafting the work or revising it critically for important intellectual content; agreement to be accountable for all aspects of the work in ensuring that questions related to the accuracy or integrity of any part of the work are appropriately investigated and resolved; ﬁnal approval of the version to be published
